# Technologies in Agronomic Biofortification with Zinc in Brazil: A Review

**DOI:** 10.3390/plants14121828

**Published:** 2025-06-14

**Authors:** Ana Beatriz Pires Silva, Lidiane Fátima Santos Borges, Fabíola Lucini, Gutierres Nelson Silva, Elcio Ferreira Santos

**Affiliations:** 1 Faculdade de Ciências Agrárias, Federal University of Grande Dourados, Dourados 79804-970, MS, Brazil; agro.anabeatriz@gmail.com (A.B.P.S.); lidianeborges300@gmail.com (L.F.S.B.); 2 Laboratório de Pesquisa em Ciências da Saúde, Universidade Federal da Grande Dourados, Dourados 79804-970, MS, Brazil; fabiolalucini10@gmail.com; 3 Federal Institute of Mato Grosso do Sul, Nova Andradina 79750-000, MS, Brazil; gutierres.silva@ifms.edu.br

**Keywords:** micronutrient deficiency, food and nutritional security, food safety, sustainable agriculture, human health

## Abstract

Zinc deficiency is a major contributor to hidden hunger, affecting billions of people worldwide, particularly in vulnerable populations. Agronomic biofortification with zinc is a promising strategy to increase both crop productivity and the nutritional quality of food, especially in countries like Brazil, where tropical soils are often deficient in this micronutrient. This review analyzes the main technologies applied in the zinc biofortification of edible crops in Brazil, including fertilizer types, application methods, doses, and the use of innovative approaches such as nano-fertilizers and biofertilizers. The results show that the foliar application of zinc sulfate at doses of 600 g ha^−1^ increased zinc concentration in grains by 25–40% without reducing crop yields. Additionally, the use of zinc nanoparticles increased wheat grain zinc content by up to 30% and biomass production, while biofertilizer application with diazotrophic bacteria raised zinc concentration in maize grains by 12.7–18.2%. These technologies demonstrate potential for enhancing zinc use efficiency and improving the nutritional quality of crops. Standardizing biofortification practices is essential to maximize their impact on food and nutritional security, contributing to the prevention of zinc deficiency in human populations.

## 1. Introduction

Zinc is an essential mineral micronutrient required in small quantities for the proper development and functioning of all living organisms [1]. In plants, this element is directly involved in cellular metabolism and is an integral part of the structure of various enzymes, being crucial for photosynthesis and carbohydrate metabolism [2]. In the human body, zinc plays a key role in several physiological and structural functions, acting as an enzymatic cofactor and participating in approximately 10% of the body’s total proteins [3,4].

Zinc deficiency has significant impacts, especially on vulnerable groups such as children and pregnant women [5,6]. In many countries, particularly in low-income regions, the prevalence of this nutritional deficiency reflects dietary inequalities and limited access to diverse diets, requiring preventive measures such as biofortification, supplementation, and food fortification [7]. Therefore, the implementation of agronomic biofortification technologies is a promising strategy to simultaneously improve crop productivity and address micronutrient deficiencies in human populations.

The deficiency of this micronutrient in humans is associated with significant adverse impacts, such as impaired growth, neurological development issues, immune dysfunction, diseases like cancer, memory disorders, respiratory diseases, and cardiovascular conditions [8,9,10]. It is a global public health issue, affecting approximately 2 billion people annually [2,11]. Inadequate zinc intake has raised significant concerns, driving interest in strategies to improve the micronutrient content of food and, thereby, benefit human nutrition and health [12].

In soils from tropical biomes, such as those in Brazil, symptoms of zinc deficiency in plants are frequently observed due to the low natural availability of the mineral [13]. Zinc is generally the most deficient micronutrient in these soils, with average levels of 64 mg/kg, although this concentration can vary depending on the soil’s parent material [14,15]. To address this limitation, various strategies have been studied and implemented, with agronomic biofortification standing out. This technique aims to increase the bioavailable nutrient content in the edible parts of plants—such as roots, leaves, and grains—through genetic improvement, agronomic practices, or nutritional management [16].

Ensuring global food security has become an increasingly urgent priority, as projections indicate that food demand could rise by 59–90% to meet the needs of an estimated 9.7 billion people by 2050 [17]. This growing demand places significant pressure on agricultural systems to increase production using the same amount of arable land [18]. In response to this challenge, many governments have implemented various programs and policies aimed at enhancing both the yield and nutritional quality of grain crops [19]. Among the strategies developed to combat nutritional deficiencies, biofortification stands out as an effective solution, particularly in regions where staple grains constitute a major portion of the diet (Harvest Plus). This approach seeks to enrich staple crops with essential nutrients, targeting populations most vulnerable to malnutrition and micronutrient deficiencies [20,21,22].

Biofortification can be achieved through agronomic practices—such as soil and foliar applications or the use of microbial inoculants—or through genetic methods, including conventional breeding, molecular techniques, and marker-assisted selection [8,22,23,24]. Both approaches aim to improve the nutrient content and bioavailability in the edible parts of crops, thereby contributing to better human nutrition. This strategy is particularly important in addressing hidden hunger, a widespread form of malnutrition that disproportionately affects women and young children, often due to an insufficient intake of key micronutrients [14]. Recently in Brazil, increasing the concentrations of essential nutrients—such as zinc, iron, selenium, and various vitamins—in commonly consumed crops was proven to be a sustainable and cost-effective strategy to enhance dietary quality and overall public health [22,25,26,27,28,29,30]. While the external application of micronutrient fertilizers is an effective practice, the efficiency of biofortification depends on the plant species used and its genetic characteristics [31].

The significance of exploring technologies in agronomic biofortification with zinc in Brazil lies in the country’s prominent role as a global agricultural producer and the ongoing challenges related to micronutrient deficiencies in its population [30,32,33,34,35]. Zinc deficiency, in particular, poses serious public health concerns, contributing to impaired immune function, stunted growth in children, and increased susceptibility to infections. In Brazil, these issues are further exacerbated by its natural availability in tropical soils, which directly affects the nutritional quality of staple crops. Implementing effective biofortification technologies not only enhances the nutrient density of commonly consumed foods but also aligns with sustainable agricultural practices aimed at improving food security, public health, and economic resilience [4,36]. Therefore, understanding and evaluating the available agronomic biofortification strategies—especially those tailored to Brazil’s diverse climatic and soil conditions—is crucial for developing scalable solutions that can benefit vulnerable populations while supporting the country’s agricultural productivity. In this context, this study aims to critically analyze the main technologies, strategies, and research advances in agronomic zinc biofortification that have been applied to edible crops in Brazil over the last decade. By systematizing current knowledge, identifying gaps, and comparing approaches, this study provides insights to support future research, technological development, and the adoption of biofortification as a strategy to improve food and nutritional security. Although focused on Brazil, this review also incorporates international perspectives to strengthen the scientific basis and global relevance of the topic. This review was organized to systematize the current knowledge of Brazilian research on this topic, highlighting advancements, gaps, and opportunities for future investigations.

## 2. Methodology

The literature review was conducted following the guidelines of the Preferred Reporting Items for Systematic Reviews and Meta-Analyses (PRISMA) [37]. The process was divided into four main stages: the identification, screening, eligibility assessment, and inclusion of studies.

In the identification stage, specific descriptors combined with Boolean operators were used to ensure comprehensive and precise search across the scientific databases PubMed, Google Scholar, and CAFe Capes, accessed between 10 and 25 September 2024. The main search terms included “agronomic biofortification”, “zinc”, and “Brazil”, and the search strategy was adapted for each database to maximize the retrieval of relevant studies. Additionally, a manual search was conducted through the reference lists of the included articles to ensure the identification of complementary studies that might not have been captured in the electronic searches.

In the screening stage, the titles and abstracts of the articles were initially reviewed to eliminate those that did not meet the basic eligibility criteria. Next, an eligibility assessment was carried out through a full reading of the articles, rigorously applying the predefined inclusion and exclusion criteria. The inclusion criteria were as follows: (i) original studies on agronomic zinc biofortification in edible plants in Brazil, (ii) articles published between 2014 and 2024, in English or Portuguese, and (iii) studies that analyzed different sources and methods of zinc application and their impact on the concentration of the mineral in the edible parts of plants. The exclusion criteria included duplicate articles, literature reviews, studies that did not focus on biofortification in Brazil, or those addressing biofortification with nutrients other than zinc.

The selection of studies was carried out in three stages: the reading of titles, reading of abstracts, and full reading of the articles. Two independent reviewers participated in each stage, and disagreements were resolved by a third reviewer. To organize the studies and eliminate duplicates, the Mendeley reference management software (version 2.98.0) was used. After selection, a standardized form was created for the extraction of relevant data, such as the source and form of the micronutrient used, applied doses, method of application, timing of application, and genotype/phenotype of the plants studied. The results were analyzed qualitatively, with an emphasis on identifying factors influencing crop agronomic performance and zinc accumulation, such as the fertilizer type, application method, dose, and environmental condition. Additionally, a narrative synthesis of the main findings was conducted, highlighting gaps in the current literature and implications for future research.

## 3. Results and Discussion

The search in the selected databases, following the strategy established in the PRISMA protocol, initially resulted in the identification of 46 articles related to the topic of agronomic zinc biofortification in Brazil. These articles were distributed as follows: 25 in Google Scholar, 13 in CAFe—Capes Periodicals, and 8 in PubMed. After removing duplicates, 43 articles were considered.

The manual screening stage, considering the previously defined exclusion factors—such as publication date (from 2014 onward), language (Portuguese or English), focus on zinc biofortification in edible plants, and location in Brazil—led to the removal of 14 articles. After the initial screening, another screening was conducted, during which articles that did not directly address zinc micronutrient biofortification, literature reviews, and studies involving non-plant organisms were removed, totaling five more articles excluded.

**Figure 1:**
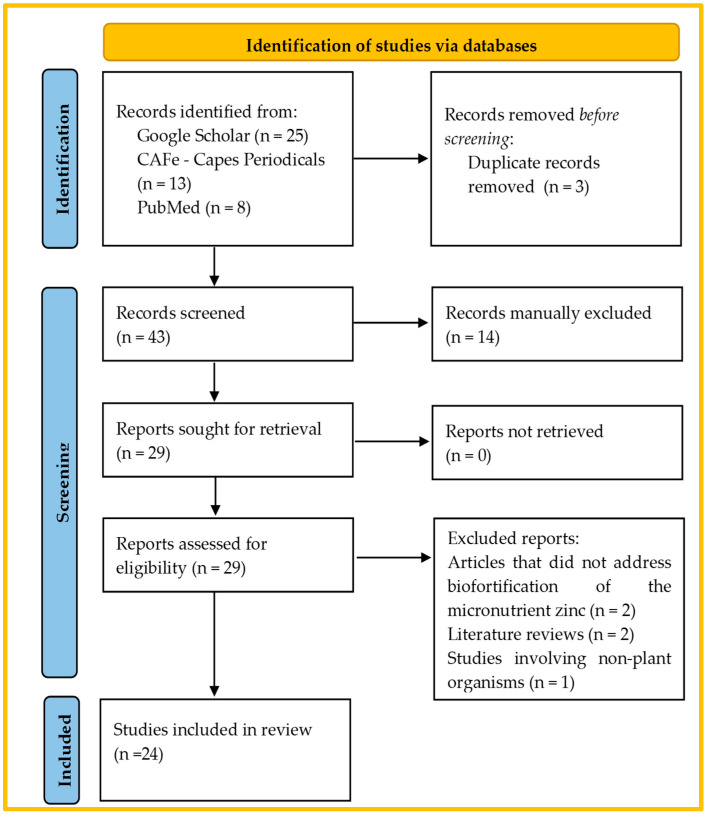
The selection process of the eligible papers based on the PRISMA 2020 flow diagram.

After screening, 24 articles were deemed eligible and included in the review. The study selection process is illustrated in the PRISMA flowchart in Figure 1.

### 3.1. Zinc Application Method

The method of zinc application is a determining factor for the success of agronomic biofortification technologies, directly impacting the concentration of the micronutrient in the edible parts of plants. While soil fertilization remains widely used, several studies indicate that foliar application is more efficient in incorporating zinc compared to the traditional method [14,23,36,38]. Kachinski [14], when investigating zinc biofortification in common beans, demonstrated that foliar application significantly increased the mineral concentration in the grains, surpassing the results obtained through soil fertilization. Additional studies support these findings: Kachinski [14], Prom-u-thai [23], Oliveira [36] and Jalal [38] observed substantial increases in zinc levels in various commercially important crops, such as cowpea, wheat, and rice, when the fertilizer was applied foliarly. In a study by [23] conducted in six countries, the foliar application of zinc alone resulted in an increase in zinc content in brown rice grains from 21.4 mg/kg to 28.1 mg/kg. Kachinski [14] also highlighted that a single foliar spray, applied during the early grain filling stage, was the most effective method to increase zinc concentration in common bean grains without negatively affecting crop yield. Similarly, Oliveira [36] reported improvements in the nutritional quality of cowpea grains in response to foliar zinc application.

In contrast, Fonseca [39], analyzing chickpea production in response to foliar and/or soil zinc fertilization and phosphorus doses, did not observe any effect on production components concerning zinc fertilization. This lack of response was attributed to the high natural soil fertility and the higher presence of organic matter.

In addition to foliar application, other forms of zinc supplementation have shown potential in different crops. Application through nutrient solutions is one of these promising strategies [40,41]. Carmona [40] in studying the osmotic conditioning of beetroot seeds, showed that enriching nutrient solutions with zinc promoted improvements in plant growth, yield, and zinc concentration in the roots, highlighting the effectiveness of this technique in biofortification. Osmotic conditioning allows for controlled seed hydration with a specific nutrient solution for a determined period of time, aiming to improve certain physiological characteristics. In this study, the seeds were immersed in 10 mL of solution with calculated amounts of Zn for the time specified in each treatment, which were 12, 16, 20, and 24 h, respectively.

Another alternative method highlighted by Mayorquin Guevara [41] involves applying zinc sulfate solutions directly to the pseudostem of banana plants. This method tripled the zinc content in the fruit compared to the control, proving to be an efficient approach for biofortifying specific crops. Tremea [42], analyzing the foliar application of zinc and iron, either individually or in combination, observed an influence on the physiological quality of white oat seeds. That is, it affected the performance of vital and physical seed functions, such as germination, accelerated aging, root length, shoot length, total length, seedling dry mass, and electrical conductivity.

These results indicate that the choice of the zinc application method should be based on the characteristics of the target crop and management conditions, ensuring the maximization of mineral absorption and its accumulation in the edible parts of the plants. The impact of different application methods is detailed in Table 1.

For conventional agriculture, the available data clearly favor the foliar application of zinc sulfate as the most efficient strategy for agronomic biofortification, especially during the reproductive stage of crops. Studies with common bean, rice, and cowpea demonstrate that a single foliar application of 600 g ha^−1^ of Zn at early grain filling significantly improves grain zinc concentrations without reducing yields, with reported increases of 25–30% in zinc content compared to soil applications [14,23,36]. This method offers a practical and cost-effective solution, particularly when the goal is the nutritional enrichment of grains or seeds.

### 3.2. Fertilizer Types, Doses, and Application Efficiency

The choice of zinc source is a crucial parameter for the success of agronomic biofortification, directly influencing the availability and absorption of the micronutrient by plants. Different zinc sources, such as sulfate, oxide, chelate, and chloride, have been evaluated in recent studies to determine their efficiency in biofortifying the edible parts of plants. According to Carmona [40], regardless of the source used, zinc sulfate or chloride, A significant increase in zinc levels in the roots of biofortified plants was observed, with an increase of more than 21% in the treatment with 30 mg/mL compared to the control. These results indicate that when applied correctly, all sources can positively contribute to nutritional enrichment.

The selection of the fertilizer type should be based not only on agronomic results but also on a cost–benefit analysis. This includes considering the price of each zinc source, as well as its chemical and physical properties, such as high water solubility, release rate in the soil, and absorption efficiency by plants [43]. Zinc sulfate (ZnSO_4_·7H_2_O) is the most widely used source in agronomic biofortification due to its high solubility in water (570 to 1000 g L^−1^ at 20 °C) and its relatively low cost [44,45]. These characteristics make it suitable for both soil and foliar applications, particularly in acidic to slightly acidic soils, which are predominant in Brazilian agriculture (pH 4.5 to 6.5) [36]. In such conditions, Zn^2+^ ions remain available and efficiently absorbed by plants. However, zinc availability is strongly influenced by soil pH: at values above 7.5, zinc tends to precipitate as zinc hydroxide (Zn(OH)_2_), significantly reducing its solubility and uptake by plants [44,45]. Under high-temperature laboratory conditions (~680 °C), zinc sulfate decomposes into zinc oxide (ZnO) and sulfur trioxide (SO_3_), but this process does not occur under field conditions [43].

In contrast, zinc chelates, such as Zn-EDTA, maintain their solubility and chemical stability across a broader pH range, remaining effective even in alkaline soils up to pH 9.0–10.0 [46,47,48]. These properties make chelated fertilizers more suitable for calcareous or high-pH soils, where conventional zinc sulfate would be less efficient. Zinc oxide, although less soluble (approximately 7.4 to 7.8 mg L^−1^ in pure water), may offer longer persistence in soil, but with lower short-term bioavailability [44,45]. Therefore, matching the zinc source with the soil pH is essential to ensure optimal nutrient availability and avoid micronutrient deficiencies.

From an economic standpoint, zinc sulfate remains the most practical and cost-effective option for large-scale applications, especially in tropical and subtropical regions where acidic soils predominate. Chelated forms, while agronomically more efficient under certain conditions, are up to four times more expensive than sulfate-based fertilizers [46,47,48], which may limit their adoption in conventional farming systems. Additionally, zinc oxide and nano-formulated sources may offer advantages in specific contexts but require further field validation. In this sense, the foliar application of zinc sulfate at a dose of 600 g ha^−1^ has consistently shown to be a technically viable and economically sustainable strategy to increase zinc concentrations in edible plant parts without compromising yield [14,36].

Therefore, the definition of the type of fertilizer to be used should be carefully planned, considering local conditions and the specific needs of each crop. From an economic and operational standpoint, zinc sulfate heptahydrate is recommended for large-scale applications due to its high solubility (570–1000 g L^−1^ at 20 °C) and lower cost compared to chelated or nano-formulated zinc. While chelates demonstrate higher bioavailability under alkaline soil conditions, their price—up to four times higher than sulfate forms—limits their widespread use in conventional agriculture. Therefore, zinc sulfate emerges as the most suitable source for foliar applications targeting both yield gains and increased zinc concentrations in edible plant parts [4,36,49,50,51].

It is also important to highlight that the zinc fraction not absorbed by plants can interact with the soil matrix, particularly in tropical soils rich in iron and aluminum oxides, leading to precipitation or adsorption processes that reduce its availability for subsequent crops. Studies suggest that only a portion of the zinc applied via soil or foliar routes is effectively translocated to the edible parts of the plants, while the remainder may accumulate in the soil or remain on the leaf surface, subject to leaching or fixation [18].

Data on the recovery efficiency of applied zinc are still limited in the Brazilian context. However, international studies indicate that zinc recovery by crops can vary widely, from 15% to 50%, depending on the chemical form, application method, and soil characteristics [52,53,54,55,56,57,58,59]. This reinforces the need for further research quantifying the difference between the amount of zinc applied and that effectively recovered in plant biomass, particularly under tropical conditions.

Over time, repeated applications without proper monitoring could lead to zinc accumulation in the soil, especially when low-solubility sources or excessive doses are used. Although zinc is an essential micronutrient, excessive accumulation may alter soil microbial activity or affect the balance of other nutrients. Therefore, agronomic biofortification strategies should consider not only immediate crop responses but also the long-term dynamics of zinc in the soil–plant system to ensure sustainability and avoid potential environmental risks [60,61,62].

It is important to clarify that, throughout this section, the term “dose” is predominantly used because most of the selected studies were conducted under field conditions, where zinc application is reported as the total amount applied per unit area (kg ha^−1^ or g ha^−1^). This metric reflects the effective quantity of the nutrient delivered to the crop. In contrast, studies carried out in hydroponic systems or using nutrient solutions describe zinc levels as “concentration” (e.g., μM or mg L^−1^), referring to the amount of zinc present in the solution. This distinction is maintained in the presentation of results to ensure consistency and comparability across different experimental approaches.

The application dose of zinc is a decisive factor in agronomic biofortification, directly influencing the zinc content in the edible parts of plants. Several studies, including those by Costa [63], Mayorquin Guevara [41], Kachinski [14] e Pascoalino [64], have demonstrated a positive correlation between the increase in the applied dose and zinc concentration in plant tissues. However, this relationship is not indefinitely linear, as excessive doses can lead to toxicity, impairing plant growth and development.

These toxic effects are associated with reduced plant growth, changes in pigment content, and decreased enzymatic activity [65]. This phenomenon aligns with Shelford’s Law of Tolerance (1913) [66], which establishes maximum and minimum tolerance limits for the development of organisms, including plants.

The analysis of dose–response relationships reinforces that 600 g ha^−1^ of zinc sulfate via foliar application is generally sufficient to achieve significant improvements in grain zinc content without causing toxicity or yield reductions in legumes and cereals. Increases beyond this dose tend to plateau, suggesting a saturation point for plant absorption systems. For soil applications, effective doses are notably higher due to losses by adsorption, as demonstrated in beetroot, where up to 6 kg ha^−1^ of zinc sulfate was needed to achieve linear increases in tuber zinc concentration [63]. Therefore, dose adjustments should prioritize application efficiency and minimize unnecessary costs.

Kachinski [14] reported a significant increase in zinc concentration in bean grains up to the dose of 600 g/ha^−1^. Beyond this dose, concentration values remained stable, showing no statistical differences, indicating the saturation point of the plant’s absorption system. Similarly, Mayorquin Guevara [41] emphasized that, in banana plants, doses exceeding certain limits did not provide additional gains in zinc concentration, reinforcing the importance of proper dose management to avoid waste and negative impacts on plant development. An analysis of the tested doses and their effects is presented in Table 2.

### 3.3. Influence of Genotypes/Phenotypes

Zinc biofortification is influenced not only by external factors, such as the type of fertilizer or application method, but also by the genetic characteristics of the plants. Both phenotype and genotype play essential roles in the process of zinc absorption and accumulation in plant tissues. Among the articles selected for this review, six studies investigated the influence of plant lines and genetic parameters on zinc biofortification.

Studies such as those by Freitas [67], Zanotti [68], Mazieiro [69] e Martins [70] emphasize the importance of selecting appropriate genotypes, as some plant lines naturally exhibit higher efficiency in zinc uptake and accumulation. This suggests the presence of specific genes that regulate micronutrient absorption from the soil and its storage in the edible parts of the plant. These genetic differences are crucial for the development of biofortified cultivars with greater nutritional efficiency. Zillio [71], analyzing the genotype x environment interaction by cultivating several common bean genotypes in different locations, found a strong influence of the genotype on zinc, phosphorus, and crude protein, with no interaction between the genotypes and the effects of the environment.

Furthermore, studies indicate that certain agronomic traits associated with biofortification exhibit high heritability, which facilitates the selection of promising genotypes [72,73]. The main genotypic effects observed are summarized in Table 3.

### 3.4. Special Fertilizers (Nanoparticles)

The application of fertilizers containing zinc nanoparticles (nano-Zn) has proven to be an emerging technology for agronomic biofortification, offering enhanced absorption and efficiency in micronutrient use. Jalal [38] demonstrated that the foliar application of zinc oxide in the form of zinc nanoparticles on wheat, during the 2019 and 2020 growing seasons, significantly increased the zinc concentration in both the aerial parts and the grains of the plant. Among the tested doses (0, 0.75, 1.5, 3, and 6 kg Zn ha^−1^), the 1.5 kg Zn ha^−1^ dose was the most effective in terms of increasing dry matter content and the zinc partitioning index. In both growing seasons, this dosage showed superior results compared to the lower doses and was similar in efficiency to the 3 kg Zn ha^−1^ dose. However, in the 2019 season, grain yield was slightly higher with the 3 kg Zn ha^−1^ dose.

The use of nanoparticles is advantageous because it provides a larger surface area and better penetration into plant tissues, resulting in higher nutrient absorption [74]. This strategy is especially promising under adverse environmental conditions, where the conventional absorption efficiency of fertilizers may be limited. Jalal [49] support this conclusion, highlighting that the application of nano-Zn improves not only the zinc content in plants but also the overall performance of the crop.

Therefore, the use of nanoparticle-based fertilizers represents a viable technology to optimize the biofortification of crops under various agricultural conditions, contributing to food security and the sustainability of the production system [49,74]. Although still under development, zinc nano-fertilizers present a promising alternative for improving both nutrient use efficiency and biofortification potential. For example, the foliar application of nano-Zn at 1.5 kg ha^−1^ in wheat effectively increased grain zinc concentration and biomass production, with a performance comparable to higher doses but reduced risks of environmental losses or toxicity [38]. Such approaches may be particularly valuable in adverse conditions or for crops with high zinc requirements, although cost and accessibility remain challenges for broad adoption in conventional systems.

### 3.5. Biofertilizers (Test with Bacteria)

The use of diazotrophic bacteria co-inoculated with zinc has proven to be a promising and sustainable approach for the agronomic biofortification of various crops, although it remains a relatively underexplored field in the literature. These biological technologies, when integrated with conventional fertilization, offer a sustainable alternative that enhances both zinc uptake and crop performance. Jalal [75] conducted an experiment using the co-inoculation of different bacterial combinations, including Rhizobium tropici, R. tropici + Azospirillum brasilense, R. tropici + Bacillus subtilis, R. tropici + Pseudomonas fluorescens, R. tropici + A. brasilense + B. subtilis, and R. tropici + A. brasilense + P. fluorescens, demonstrating positive results in grain biofortification. The study showed a significant increase in zinc content in the soil, leaves, shoots, and grains. Furthermore, it improved the zinc partitioning index, reflecting favorable agronomic characteristics such as greater plant height, increased shoot dry mass, and higher grain yield.

It is important to highlight that the use of diazotrophic bacteria in co-inoculation strategies, while promoting zinc uptake, may also influence the mobilization and availability of other soil nutrients such as phosphorus, selenium, and micronutrients [22,26,27]. This occurs through mechanisms like organic acid production, rhizosphere acidification, or siderophore release, which can alter soil nutrient dynamics [18]. Although current studies report positive effects on crop yield and zinc concentration in grains, they are predominantly short-term trials [75,76]. The long-term impacts of the repeated use of such inoculants remain uncertain, particularly regarding the potential risk of gradual soil nutrient depletion. The continuous extraction of mobilized elements without proper nutrient replenishment could compromise soil fertility over time. Therefore, further long-term studies are needed to evaluate the sustainability of this practice and guide nutrient management strategies to avoid soil exhaustion in biofortification systems using microbial inoculants.

Another relevant aspect of this study was its impact on the daily zinc intake of the Brazilian population, indicating that the use of bacteria could have direct implications for nutritional security. Subsequent trials reinforced these results. Jalal [76], investigating biofortification in corn, and Jalal [38], studying wheat, observed consistent increases in plant height, dry matter, and grain yield. Moreover, a substantial increase in zinc concentration was reported in the leaves, shoots, and grains, as well as in the accumulation of the mineral in the aerial parts of the plants.

Therefore, the use of biofertilizers based on diazotrophic bacteria represents an effective and sustainable alternative for optimizing the biofortification of crops such as beans, corn, and wheat, ensuring improvements in nutritional quality and agricultural yield. The results of this practice are presented in Table 4.

The integration of diazotrophic bacteria co-inoculated with zinc represents a sustainable strategy with dual benefits: enhanced zinc uptake and improved plant growth. In maize, this approach increased zinc concentration in grains by 12.7% to 18.2% across two seasons while improving agronomic traits such as plant height and yield [76]. Although the technology requires further validation for different crops and environments, it offers a promising pathway for reducing dependence on chemical fertilizers while achieving biofortification goals [77,78,79,80].

### 3.6. Health Impacts

The zinc biofortification of crops is an effective strategy to increase the mineral content in plants, benefiting regions where zinc nutritional deficiency is common and represents a public health issue. The consumption of zinc-rich foods can prevent a range of conditions related to this micronutrient deficiency, such as impaired child growth, immune system suppression, and slow wound healing [81].

Sousa [82] emphasize that agronomic biofortification can have a direct impact on public health by increasing zinc levels in food crops. In their experiments with lettuce, foliar zinc application resulted in elevated zinc levels in the edible parts of the plant, demonstrating the potential of this technique to improve zinc intake through the diet. The increases in zinc levels in the leaves, compared to the control (0 g/ha), with the application of 200 g/ha^−1^ of sulfate, 562 g/ha^−1^ of oxide, and 72.5 g/ha^−1^ of zinc chelate were 206, 678, and 106 mg/kg of zinc, respectively.

An important aspect when discussing strategies to combat human zinc deficiency is comparing the effectiveness and cost of agronomic biofortification with direct supplementation, such as the use of zinc glutamate tablets. While supplementation offers a rapid and targeted correction of acute zinc deficiency, especially in vulnerable populations, it presents limitations such as cost, the need for continuous health system distribution, adherence challenges, and potential side effects at higher doses. On the other hand, biofortification through zinc-enriched food provides a sustainable, long-term approach that integrates naturally into the daily diet, reducing dependency on healthcare services [83,84,85].

Economic analyses suggest that the cost of zinc supplementation (e.g., zinc glutamate tablets) can range from USD 2 to 4 per person per year, depending on the program scale and logistics, while agronomic biofortification costs are incorporated into existing agricultural practices, potentially reaching a much broader population at a lower marginal cost per individual. Moreover, biofortification has the added benefit of improving the nutritional quality of staple foods without requiring changes in eating habits [84,86]. However, it is important to recognize that both strategies are complementary: supplementation is essential for treating severe cases, while biofortification acts as a preventive and population-wide measure. Further studies evaluating the cost-effectiveness of both strategies under Brazilian conditions are recommended to guide public health policies.

Biofortification through agronomic techniques, such as the selection and breeding of plant varieties with higher zinc absorption capacity, provides a sustainable and long-term approach to mitigating nutritional deficiencies. This approach can have a significant impact on human health, particularly in low-income areas and vulnerable populations, where access to supplements or fortified foods is limited [87,88,89,90,91]. Although supplementation with zinc glutamate tablets is effective in correcting acute deficiencies, biofortification presents itself as a more accessible long-term alternative, naturally integrating into the diet without the need for medical interventions. Furthermore, the presence of bioactive compounds in biofortified foods can increase zinc bioavailability, making this strategy even more efficient in promoting health and reducing nutritional insecurity.

Taken together, these findings enable the formulation of preliminary practical guidelines for zinc agronomic biofortification in Brazilian conventional agriculture. The use of zinc sulfate heptahydrate via foliar application at doses of 600 g ha^−1^ during the reproductive stage stands out as a technically viable and cost-effective practice to increase zinc concentration in grains by 25–40% and achieve modest yield gains of 5–15%, depending on the crop and conditions. Standardizing such protocols can facilitate comparability between studies and accelerate the adoption of biofortification strategies aimed at mitigating hidden hunger [36,92,93].

## 4. Future Perspectives and Strategies

The data presented in this review highlight the complexity and diversity of practices involved in zinc biofortification in Brazil. Various zinc sources—including sulfate, oxide, chelate, and chloride—combined with different application methods and dosage levels have been extensively studied. However, this heterogeneity complicates direct comparisons between studies and hinders the identification of the most effective and widely applicable strategies.

The future success of agronomic zinc biofortification relies on overcoming current challenges related to methodological variability, environmental sustainability, and the translation of research findings into large-scale agricultural practices that improve human nutrition.

A key priority is the standardization of experimental protocols. This involves developing harmonized guidelines that define essential aspects such as zinc sources (e.g., zinc sulfate, zinc oxide, nanoparticles), application methods (soil, foliar, or fertigation), optimal doses, timing relative to crop growth stages, and the selection of target crops and edible plant parts for zinc quantification. Standardization will enhance comparability across studies, reduce variability caused by differing methodologies, and generate more robust data to inform practical recommendations for farmers, industries, and policymakers.

Achieving standardization requires collaborative efforts among research institutions, agricultural agencies, and international programs. These partnerships should work to establish guidelines tailored to various crops, soil types, and climatic conditions—particularly in tropical regions like Brazil, where soil characteristics pose unique challenges to zinc availability and uptake.

Future strategies should also include comprehensive economic feasibility studies to evaluate the cost-effectiveness of agronomic biofortification compared to alternative interventions such as zinc supplementation or industrial food fortification. These studies are essential for supporting public policies and positioning biofortification as a sustainable, large-scale strategy to combat hidden hunger.

Environmental sustainability must be a core focus of future research. Continuous zinc fertilizer application may lead to soil accumulation, environmental contamination, and adverse effects on soil microbial communities. Long-term field studies are needed to monitor these impacts and ensure the safe and responsible adoption of biofortification technologies [60,61,62,94,95].

Emerging technologies, such as nano-fertilizers and microbial inoculants (biofertilizers), offer promising potential to improve zinc use efficiency and minimize environmental losses. However, further research is necessary to validate their field effectiveness, assess potential risks, and determine their economic viability for large-scale application [38,44,46,48,49,74,96,97].

Additionally, integrating plant breeding programs to select or develop crop genotypes with enhanced zinc uptake and accumulation capacity could significantly increase the efficiency of agronomic biofortification. Combining genetic improvements with optimized agronomic practices will maximize the impact of this strategy.

Future perspectives must also embrace multidisciplinary and intersectoral approaches that bridge agriculture, nutrition, health, economics, and environmental sciences. Strengthening international collaboration and aligning biofortification efforts with global health initiatives—such as the Sustainable Development Goals (SDGs)—will be essential for tackling hidden hunger and advancing global food and nutritional security.

Overall, agronomic zinc biofortification holds significant promise as a sustainable strategy to boost crop productivity and address global nutritional challenges. Its success will depend on interdisciplinary collaboration, technological innovation, and the development of standardized practices that ensure its scalability and lasting impact.

## 5. Conclusions

The adoption of agronomic biofortification technologies represents a feasible strategy to enhance global food and nutritional security, particularly in regions with zinc-deficient soils. Agronomic zinc biofortification not only improves crop productivity but also enhances the nutritional quality of food, offering a sustainable solution to address widespread human zinc deficiency.

This review highlights that the foliar application of zinc sulfate—especially during the reproductive stage of crops—remains the most effective and economically viable method for increasing zinc concentrations in edible plant parts without compromising yield. However, the success of biofortification programs depends on several critical factors, including the choice of zinc source, application method, dose optimization, and crop genotype. Emerging technologies, such as nano-fertilizers and microbial inoculants, show promising potential to improve zinc use efficiency but require further validation under field conditions.

Importantly, when compared to direct zinc supplementation strategies, such as Zn glutamate tablets, agronomic biofortification offers a more cost-effective and sustainable long-term approach. It naturally integrates essential micronutrients into daily diets, making it especially beneficial for low-income populations. Nevertheless, these strategies are complementary: supplementation remains crucial for addressing acute deficiencies, while biofortification serves as a population-level intervention to reduce hidden hunger.

Future research should prioritize the standardization of biofortification protocols, the assessment of long-term environmental impacts, and the exploration of biofortification’s role in strengthening global food and nutritional security. Expanding multidisciplinary studies that integrate agricultural, nutritional, environmental, and economic perspectives will be essential to maximize the impact of zinc biofortification on human health and to support progress toward achieving the Sustainable Development Goals (SDGs).

## Figures and Tables

**Table 1:** Zinc application method.

	Culture	Test Conducted	Application Method	Form of Applied Zinc	Zn Concentration	Application Stage	Effect	Side Effects	Ref.
1	Common bean	In the field	Soil: seeding furrow vs. leaf: backpack sprayer	Zinc sulfate heptahydrate	Soil: 4 kg ha^−1^ incorporated into NPK fertilizer with 1% Zn; leaf: 0, 300, 600, 900, 1200 and 1500 g ha^−1^	Soil: planting; leaf: R6, R8 e R9	The single foliar application of 600 g ha of Zn at the early grain filling stage proved to be the most effective method for improving Zn concentration in the grains, without affecting yield. The agronomic biofortification of bean grains with Zn was more efficient with foliar Zn application than with soil Zn fertilization.	Soil fertilization with zinc resulted in an average increase of 20% to 30% in the concentration of total amino acids and storage proteins, such as albumin, globulin and glutelin, compared to treatments without zinc application.	[14]
2	Cowpea	Greenhouse	Leaf CO_2_ pressurized backpack sprayer	Zinc sulfate heptahydrate	0 and 600 g ha^−1^	Full flowering 43 days after sowing (DAS) in the 1st year and 42 DAS in the 2nd year	All genotypes evaluated show zinc enrichment in the grains in response to the foliar application of the micronutrient in the form of zinc sulfate.	There was a significant increase in the concentrations of storage proteins, total free amino acids, sucrose and total sugars.	[36]
3	Wheat	In the field	Leaf: hand pump	Nano-Zn and bacteria: *Azospirillum brasilense*, *Bacillus subtilis e Pseudomonas fluorescens*	0, 0.75, 1.5, 3 and 6 kg ha^−1^	Profiling and grain filling	The foliar application of Nano-Zn increased N, P and Zn concentrations in the plant and grain, in addition to helping wheat growth and yield.	There was an increase in the concentrations of zinc, nitrogen and phosphorus in both the aerial part and the wheat grains. Inoculation with Pseudomonas fluorescens increased phosphorus concentration in grains by up to 32.2% compared to the control.	[38]
4	Rice	In the field	Leaf: hand pump	Zinc sulfate heptahydrate	0.5% ZnSO_4_ 7H_2_O in spray solution. The same concentration was used in a micronutrient cocktail with iodine, iron and selenium	Panicle initiation and early stages of grain milk	The zinc content in brown rice grains increased with the foliar zinc alone and micronutrient cocktail treatments. The increase in zinc content was from 21.4 mg/kg to 28.1 mg/kg with the application of the nutrient and 26.8 mg/kg with the micronutrient cocktail solution, applied via foliar application.	Iodine and selenium showed significant increases. The iron concentration in the grains was not significantly affected by the application of the micronutrient cocktail. There were no significant results for protein levels.	[23]
5	Beet	Greenhouse	Nutrient solution: seeds immersed in different concentrations of zinc	Zinc sulfate and chloride	0, 10 and 30 mg mL^−1^	Seed priming	The application of zinc, mainly as a sulfate, affected all the parameters evaluated, such as fresh mass (an increase of 70 and 100 g per plant with 10 mg/mL of Zn) and dry root mass, photosynthesis, and zinc concentration in the root (121 and 42 mg/kg) in the years 2015 and 2016, respectively.	The study did not measure protein and amino acid concentrations.	[36]
6	White oats	In the field	Leaf: backpack sprayer	Zinc sulfate	0, 1000, 2000 and 4000 g ha^−1^	Exposed inflorescence, at the beginning of grain filling	The application of zinc, either alone or in combination with iron via foliar spraying, influenced the physiological quality of white oat seeds. This term refers to the set of attributes that determine the seeds’ potential to germinate and produce vigorous seedlings capable of establishing successfully under varying environmental conditions. Depending on the applied dose and the parameter evaluated—such as germination rate, seedling growth, or membrane integrity—the effects observed were either beneficial or detrimental.	The foliar application of iron and zinc, alone or combined, positively influenced the chemical composition of seeds, including protein and nitrogen content. The use of iron and zinc contributed to improving the nutritional quality of the seeds, resulting in greater vigor and metabolic efficiency during seedling development.	[42]
7	Banana	In the field	Nutrient solution: injections of solutions with a 9 cm needle to reach the central axis of the pseudostem.	Zinc sulfate	10.83 g/L (1%), 21.66 g/L (2%) and 43.32 g/L (4%)	15 days before harvesting the fruits	It was possible to biofortify the banana plant by injecting a solution containing 20 and 40 g/L of zinc sulfate into the pseudostem, tripling the zinc content in the fruits compared to the control, with levels of 3.66 to 3.39 mg/100 g.	Protein levels were not evaluated. However, there was an increase in the soluble solids/acidity ratio, which may improve the perception of sweetness.	[41]
8	Chickpea	In the field	50% soil and 50% leaf, and 100% soil. Soil: sowing; sheet: hand sprayer	Zinc sulfate	2 kg/ha	Sowing and flowering	The zinc fertilization of chickpeas did not influence the crop’s production components.	Zinc played a complementary role in nutritional management, increasing the efficiency of phosphorus use at lower doses.	[39]

**Table 2:** The influence of the doses used.

	Culture	Test Conducted	Application Method	Form of Applied Zinc	Zn Concentration	Application Stage	Effect	Side Effects	Ref.
1	Beet	In the field	Nutrient solution: fertigation	Zinc sulfate	0, 1.5, 3.0, 4.5 and 6.0 kg ha^−1^	23 days after sowing (2019) and 29 days (2021)	In the 2021 experiment, increasing zinc doses promoted the biofortification of beetroot, as it demonstrated a linear increase in the zinc levels contained in the plant’s tuberous root.	There was no evaluation of amino acids and proteins.	[63]
2	Banana	In the field	Nutrient solution: injections of solutions with a 9 cm needle to reach the central axis of the pseudostem	Zinc sulfate	10.83 g/L (1%), 21.66 g/L (2%) and 43.32 g/L (4%)	15 days before harvesting the fruits	It was possible to biofortify the banana plant by injecting a solution containing 20 and 40 g/L of zinc sulfate into the pseudostem, tripling the zinc content in the fruits compared to the control, with levels of 3.66 to 3.39 mg/100 g.	Protein levels were not evaluated. However, there was an increase in the soluble solids/acidity ratio, which may improve the perception of sweetness.	[41]
3	Common bean	In the field	Soil: seeding furrow vs. leaf: backpack sprayer	Zinc sulfate heptahydrate	Soil: 4 kg ha^−1^ incorporated into NPK fertilizer with 1% Zn; leaf: 0, 300, 600, 900, 1200 and 1500 g ha^−1^	Solo: planting; leaf: R6, R8 and R9	The single foliar application of 600 g ha of Zn at the early grain filling stage proved to be the most effective method for improving Zn concentration in the grains, without affecting yield. The agronomic biofortification of bean grains with Zn was more efficient with foliar Zn application than with soil Zn fertilization.	Soil fertilization with zinc resulted in an average increase of 20% to 30% in the concentration of total amino acids and storage proteins, such as albumin, globulin and glutelin, compared to treatments without zinc application.	[14]
4	Wheat	In the field	Nutrient solution: fertigation	Zinc sulfate	0.15 μM and 2.25 μM	Weekly: from transplanting until plant maturity	The zinc content in wheat grains more than doubled when Zn and N supplies were increased in both genotypes evaluated.	Zinc and nitrogen supply significantly influenced grain zinc concentration, while protein and amino acid content were correlated with zinc uptake and translocation in plants.	[64]

**Table 3:** Impact of genotypes/phenotypes.

	Culture	Test Conducted	Application Method	Form of Applied Zinc	Zn Concentration	Application Stage	Effect	Side Effects	Ref.
1	Cowpea	Laboratory	No information	No information	No information	No information	Analyzing 100 cowpea genotypes, it was observed that the zinc content of the grains varied from 2.35 to 4.57 g/100 g, with an average of 3.31 mg/100 g. Among the other criteria evaluated, the grains of the MNC11-1023E-28 lineage presented a better nutritional quality profile, showing potential as a food to meet consumer demands for reversing iron, zinc and protein deficiencies.	The article focuses on the analysis of iron, zinc and protein contents in different cowpea genotypes, using laboratory techniques such as X-ray fluorescence spectrometry for iron and zinc and the Kjeldahl method for proteins.	[67]
2	Common bean	Laboratory	No information	No information	No information	No information	Analyzing the diversity of the mineralogical composition in 40 common bean genotypes with a focus on the selection of promising parents in the formation of biofortified cultivars, average zinc levels between 2.8 and 4.6 mg/100 g were found. In this study, the minerals studied presented a heritability index of 80%, which suggests a high possibility of success in the selection of genotypes.	The study focuses on the analysis of the mineralogical composition of bean genotypes.	[68]
3	Common bean	In the field	No information	No information	No information	No information	The study aimed to evaluate 140 genotypes to identify elite lines that combine high levels of zinc and iron in the grains, along with good adaptability (the ability to maintain productive and nutritional performance in different environments), stability (desirable levels of iron and zinc do not vary drastically between the tested environments), and agronomic potential (the plant’s maximum genetic ability to express desirable traits). The heritability of zinc ranged from 41.7% to 95.7%, and the genetic variation coefficient ranged from 8.51% to 9.04%, indicating favorable conditions for nutrient selection.	The article addresses the application of zinc in the context of the genetic improvement of common beans to increase the iron and zinc contents in the grains.	[73]
4	Common bean	In the field/laboratory	No information	No information	No information	No information	Evaluating genetic parameters in four common bean populations, characteristics such as first pod insertion, grain yield and zinc concentration in the grain showed high heritability, showing that it is possible to select lines biofortified with zinc and with high agronomic performance through line selection.	No information	[72]
5	Common bean	In the field	No information	No information	No information	No information	Zinc concentration ranged from 2.03 to 3.60 mg/100 g in dry matter. The concentrations of K, P and Zn showed the greatest contribution to the genetic dissimilarity of the evaluated genotypes. Five lines presented zinc concentrations in the grain above 31 mg/kg, which is considered a high value.	The paper focuses mainly on the evaluation of genetic dissimilarity between common bean lines.	[69]
6	Cowpea	In the field	No information	No information	No information	No information	Evaluating the characteristics of 24 cultivars, it was observed that grain color and size did not influence the iron, zinc and protein contents in the grains.	The focus of the study is on the analysis of zinc concentrations in cowpea grains, using the nitric–perchloric digestion method and atomic absorption spectrophotometry to determine mineral contents.	[70]
7	Common bean	In the field	No information	No information	No information	No information	Evaluating the influence of different cultivation environments on 26 bean genotypes, it was observed that in relation to the levels of zinc, phosphorus and crude protein, there was no interaction between the genotypes and the environments.	The article explores the variability in protein and micronutrient contents, such as iron and zinc, in different bean genotypes grown in different locations: iron content in beans ranges from 116 mg/kg to 216 mg/kg, and crude protein content varies significantly between genotypes and growing locations.	[71]

**Table 4:** Biofertilizers (test with bacteria).

	Culture	Test Conducted	Application Method	Bacteria Used	Form of Applied Zinc	Zn Concentration	Application Stage	Effect	Side Effects	Ref.
1	Bean	In the field	Soil: surface of the soil	*Rhizobium tropic*, *Azospirillum brasilense*, *Bacilo subtilis e Pseudomonas fluorescens*	Zinc sulfate	0 and 8 kg ha^−1^	V1 and V2	The Zn concentration in the common bean plant and grain and the Zn concentration after harvesting the crop were improved.	The study did not measure protein and amino acid concentrations.	[75]
2	Corn	In the field	Soil: residual fertilization	*Azospirillum brasilense*, *Bacilo subtilis*, *e Pseudomonas fluorescens*	Zinc sulfate	0 and 8 kg ha^−1^.	V1 and V2	The insertion of the first productive ear, plant height, shoot dry matter, grain yield and hundred-grain weight increased. The Zn content in the leaves increased significantly. There was an increase of 15.2 and 15.7% in the Zn concentration in the shoot in the 2019–2020 and 2020–2021 harvests and of 12.7 and 18.2% in the Zn concentration in the grains.	The study did not measure protein and amino acid concentrations.	[76]
3	Wheat	In the field	Leaf: hand pump	*Azospirillum brasilense*, *Bacillus subtilis e Pseudomonas fluorescens*	Nano-Zn	0, 0.75, 1.5, 3 and 6 kg ha^−1^	Profiling and grain filling	The concentrations of zinc (Zn), nitrogen (N) and phosphorus (P) in the shoots and grains of wheat were significantly influenced. The dry matter of the wheat shoot increased.	There was an increase in the concentrations of zinc, nitrogen and phosphorus in both the aerial part and the wheat grains. Inoculation with Pseudomonas fluorescens increased phosphorus concentration in grains by up to 32.2% compared to the control.	[38]
